# Specific risk factors for heart-lung transplantation

**DOI:** 10.1016/j.jhlto.2025.100389

**Published:** 2025-09-17

**Authors:** Justin Issard, Jérôme Le Pavec, Gaelle Dauriat, Estibaliz Valdeolmillos, Sébastien Hascoet, Jean Noel Andarelli, Sylvain Diop, Thibaut Genty, Laurent Savale, Olaf Mercier, Elie Fadel

**Affiliations:** a Inserm UMR_S 999 (Pulmonary Arterial Hypertension: Pathophysiology and Therapeutic Innovations), Paris-Saclay University, Le Plessis-Robinson, France; b Thoracic Surgery and Heart-Lung Transplantation, Thoracic Department, Marie Lannelongue Hospital, Paris-Saclay University, Le Plessis-Robinson, France; c Pulmonology and Lung Transplantation, Thoracic Department, Marie-Lannelongue Hospital, Inserm UMR_S 999 (Pulmonary Arterial Hypertension: Pathophysiology and Therapeutic Innovations), Paris-Saclay University, Le Plessis-Robinson, France; d Department of Congenital Heart Diseases, M3C Referral Center for Complex Congenical Heart Diseases, Marie Lannelongue Hospital, Paris-Saclay University, Le Plessis Robinson, France; e Interventional Radiology, Thoracic Department, Marie Lannelongue Hospital and Paris Saclay University, Le Plessis Robinson, France; f Department of Anesthesiology and Intensive Care, Marie Lannelongue Hospital, Paris-Saclay University, Le Plessis Robinson, France; g Department of Respiratory and Intensive Care Medicine, Assistance Publique-Hôpitaux de Paris (AP-HP), ERN-LUNG, Pulmonary Hypertension National Referral Center, Bicêtre University Hospital, Inserm UMR_S 999 (Pulmonary Arterial Hypertension: Pathophysiology and Therapeutic Innovations), Paris-Saclay University, Le Kremlin-Bicêtre, France

**Keywords:** heart-lung transplantation, Eisenmenger syndrome, survival, specific risk determinant, congenital heart disease

## Abstract

**Background:**

Heart-lung transplantation (HLTx) has become increasingly rare, with fewer than 50 procedures performed annually worldwide. This decline reflects evolving indications, notably the predominance of Eisenmenger syndrome (ES) complicating congenital heart disease (CHD). HLTx remains a complex procedure associated with significant perioperative and long-term risks, particularly in this patient population.

**Methods:**

This review synthesizes current literature and registry data to identify specific risk factors associated with HLTx in its modern indications. It examines changes in patient selection, timing of listing, perioperative challenges, and postoperative outcomes, with a focus on ES and CHD-related cases.

**Results:**

HLTx candidates often present with prior thoracic surgeries, polycythemia, systemic arterial collaterals, and multi-organ dysfunction, all contributing to increased surgical complexity and bleeding risk. Long waitlist times and donor shortages further complicate management. Despite these challenges, recent data from expert centers show improved early survival, with 1-year survival rates exceeding 85%. HLTx may offer protective effects against chronic graft dysfunctions such as bronchiolitis obliterans syndrome and coronary artery vasculopathy compared to isolated organ transplants.

**Conclusions:**

HLTx remains the treatment of choice for select patients with complex cardiopulmonary disease, particularly ES with CHD. Optimizing outcomes requires early referral, careful risk stratification, and management in high-volume expert centers. Advances in surgical techniques and perioperative care have improved survival, but HLTx continues to demand multidisciplinary expertise and individualized patient assessment.

## Background

After the first successful heart-lung transplantation (HLTx) in a human in 1981,^1^ the number of HLTx procedures increased in both children and adults to a peak of about 300 worldwide in 1989.^2^ The number then declined gradually, and only 59 procedures were done worldwide in 2017, with an average of 1 case per year in each center.^2^ A major reason for this decline is that the indications for HLTx have changed over time, as detailed below.^3^ Giving the heart and lungs to the same recipient may seem challenging, given the severe shortage of donor organs. In patients with pulmonary hypertension (PH) and severe right ventricular failure, perioperative veno-arterial extracorporeal membrane oxygenation (VA-ECMO) is increasingly used to allow dual-lung transplantation (DLTx) instead of HLTx.^4^ The shortage of donors is also leading to greater use of DLTx combined with intracardiac repair of congenital heart disease (CHD). A retrospective study reported the results of DLTx and CHD repair in 33 children with PH.^5^ Hospital mortality was 25.7%, similar to the 31.3% value in the 16 patients managed with HLTx. More recently, we reported excellent outcomes of DLTx followed 3 to 6 months later by percutaneous closure of an atrial septal defect in selected patients with Eisenmenger syndrome (ES).^6^ Finally, another reason for the decline in HLTx procedures is concern about greater risks to patients compared to DLTx. The objective of this review is to discuss specific risks associated with HLTx used in its current indications.

## Changes in indications for heart-lung transplantation

The changes in HLTx indications over time reflect improvements in diagnostic strategies, perioperative care, and long-term outcomes. Initially, the main indication was end-stage pulmonary disease with secondary cardiac dysfunction, notably due to severe cystic fibrosis or idiopathic pulmonary arterial hypertension, requiring combined organ replacement.^7^ Complex CHD, particularly with ES, was then added to the indications. Data for 1990-2020 at a single center in Spain showed that the main indications for HLTx shifted toward CHD and ES and away from chronic obstructive pulmonary disease, cystic fibrosis, and idiopathic pulmonary arterial hypertension, which became indications for DLTx.^8^ Data from the 1990s established that right ventricular dysfunction reversed rapidly after DLTx in appropriately selected patients and provided similar early and long-term outcomes to those seen after HLTx, notably in patients with end-stage PH.^3^ ISHLT (International Society of Heart and Lungs Transplantation) registry data consistently show CHD as now being the main indication for both pediatric and adult HLTx, with a concurrent decrease in pulmonary-only diseases.^2^ Similarly, the United Network Organ Sharing (UNOS) registry evidences a steady decline in HLTx correlating with tighter patient selection and increased efficacy of isolated organ transplants (UNOS, 2023). Nonetheless, HLTx remains the treatment of choice in highly selected patients with end-stage cardiopulmonary diseases such as ES complicating congenital heart disease (CHD), failed CHD repair, uncorrectable CHD, and severe left ventricular failure. A recent study demonstrated improved survival after HLTx with current indications and management strategies.^9^

## Timing of listing for HLTx

Transplantation is typically considered in patients without contraindications and an expected survival of no more than 2 years with optimal medical therapy.^10^ However, accurately predicting survival time without transplantation is difficult. Notably, in patients with complex CHD, in whom HLTx is the preferred procedure, deciding whether and when to list is particularly challenging given the often-prolonged survival without transplantation, which may be improved by antihypertensive therapies. Risk scores estimating mortality in PH do not perform well in patients with ES,^11^ and neither does the pulmonary vascular resistance value.^12^ Factors that govern listing decisions include cardiac anatomy, hemodynamic status, general patient condition, and declining quality of life related to worsening cardiopulmonary failure requiring increasing hospital admissions. Listing criteria include New York Heart Association class III or IV right ventricular failure in a patient receiving optimal medical treatment, with a cardiac index <2 liter/min/m² and right atrial pressure >15 mm Hg. Hemoptysis or pulmonary artery dissection may require earlier listing.

## Long waitlist times for HLTx

Donor shortage is particularly acute for HLTx, resulting in unpredictable waitlist times of 1 to 36 months.^12^, ^13^ Longer waitlist times are associated with higher waitlist mortality. Among HLTx candidates in France in 2011-2016, only 33% were transplanted within 12 months after listing and 32% were delisted due to death or severe clinical deterioration.^14^ Changes in graft-allocation rules in the United States and Europe have decreased the availability of heart-lung blocks. Notably, because HLTx is a rare procedure, potential heart-lung-block donors are often not included in allocation score systems.^15^ Another obstacle is HLA-antibody (Human Leucocyte Antigens) development in donors due to blood transfusions during surgeries. Size-matched grafts are often difficult to obtain for patients with CHD, given their smaller body size compared to other recipients.^16^ One consequence of long waitlist times is that physicians must be prepared to provide adequate support for long periods, to decrease the high waitlist mortality. Early referral for transplant evaluation before respiratory-failure onset may shorten the waitlist time and leave less time for progression of the underlying pathology, thereby reducing waitlist mortality. In a retrospective study, waitlist mortality at 1, 3, and 12 months was 14.4%, 21.9%, and 34.2%, respectively.^12^ To decrease waitlist time and mortality, a national high-priority allocation program for HLTx was implemented in France in 2007. The results were better survival of patients with PH listed for DLTx or HLTx and a higher cumulative incidence of transplantation.^12^, ^13^

## Other organ failures: Liver and kidney

Renal failure predicts mortality in patients with heart failure.^17^ Over time, CHD can affect the function of organs other than the heart. In ES, erythrocytosis with blood hyperviscosity is associated with glomerulopathy and with tubular and interstitial kidney disease.^18^ About two-thirds of adults with CHD and ES had renal dysfunction, which was associated with higher mortality. With the introduction of PH-specific pharmacotherapy, patients may seem clinically asymptomatic but may nevertheless be developing complications such as cirrhosis due to chronic hepatic congestion, precluding HLTx.^19^ Liver function must be assessed before listing and any liver dysfunction must be treated early to avoid massive intraoperative bleeding.^12^, ^13^ When selecting patients, the frequent improvement in other organ failures after the procedure must be borne in mind.

## Polycythemia and exchange transfusion

ES, a major indication of HLTx, is associated with hypoxemia, which induces polycythemia as a compensatory mechanism.^20^ Polycythemia can cause renal failure during cardiopulmonary bypass (CPB) and increases the risk of coagulopathy, a known risk factor for postoperative complications. Intraoperative acute normovolemic hemodilution is a protective strategy in which blood is exchanged for colloids until the hemoglobin drops to 14.5 g/dl and the hematocrit to 45%.^21^ This procedure should be performed before CPB initiation. Chronic hypoxemia induces erythrocyte abnormalities that increase the risk of renal failure during CPB and the risk of coagulopathy. Combining normovolemic hemodilution with exchange transfusion can treat both the polycythemia and the erythrocyte abnormalities.^22^, ^23^

## Hypertrophic systemic bronchial and nonbronchial artery development

**Figure 1:**
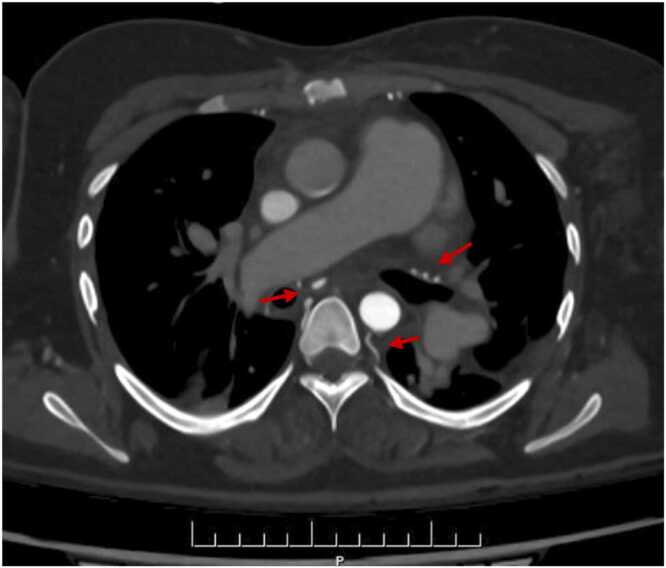
Computed tomography angiogram of the chest showing hypertrophic systemic bronchial and nonbronchial arteries in a patient with Eisenmenger syndrome complicating an uncorrected ventricular septal defect.

ES is characterized by obliterative remodeling of the pulmonary vascular bed, pulmonary microvascular changes, endothelial damage, vasoconstriction, and in-situ thrombosis.^24^ These changes trigger an inflammatory response and neo-angiogenesis with the development of bronchial and nonbronchial aortopulmonary collaterals from the subclavian, intercostal, axillary, and internal mammary arteries, as well as the infra-diaphragmatic branches of the inferior phrenic, left gastric, and celiac axis (Figure 1).^25^ These collaterals are subjected to high systemic arterial pressures, inducing a risk of bleeding during surgery.^25^ They develop considerably in CHD with extreme pulmonary artery stenosis, to maintain blood flow to the lungs. Despite careful preoperative angiographic assessment and the introduction of technical improvements to reduce the bleeding risk,^26^ bleeding is among the main complications of HLTx, notably for ES. In a 1995 study, 20% of patients required repeat surgery for hemostasis.^27^ A database study of HLTx (*n* = 57) or lung transplantation plus heart repair (*n* = 6) done for ES in 1985-2012 in the Nordic region found that one-third of patients required reoperation within the first week, including 95% for bleeding.^28^ The transfusion of large volumes of blood products required to manage perioperative bleeding may precipitate primary graft dysfunction.

## Previous thoracic surgery

Due to surgical advances in palliative surgery, patients with complex CHD (i.e., tetralogy of Fallot, transposition of the great arteries, and pulmonary atresia) often undergo several surgical procedures during childhood to delay transplantation.^29^ Thus, most HLTx candidates have a history of cardiovascular surgery, which results in the development of adhesions, anatomical alterations, and collateral blood vessels. Postoperative adhesions are very different from those observed in patients without CHD, being dense and highly vascularized, with the vascular remodeling described above. Arterial collaterals arising from the intercostal, internal thoracic, and axillary arteries penetrate the lung parenchyma, enlarge over time, and are difficult to control during recipient pneumonectomy. The result is considerable blood loss, a longer CPB time, hemodynamic instability with a need for transfusion of multiple blood products, and in some cases repeat surgery.^5^ CPB carries a risk of coagulation disorders, decreased brain perfusion, acute kidney injury, and arrhythmia.^30^ Dissection of the dense adhesions can damage the phrenic nerve, vagus nerve, or lymphatics. Phrenic nerve dysfunction has been reported in over 40% of patients undergoing HLTx.^30^ One-third of the patients with phrenic nerve dysfunction died, chiefly of pulmonary complications, and only 11.1% fully recovered their forced vital capacity. Complications of phrenic nerve damage include longer times on invasive mechanical ventilation (iMV) and in the ICU (Intensive Care Unit), which in turn are associated with higher morbidity and mortality. Damage to the vagus nerve, which runs near the tracheal anastomosis, may result in gastroparesis, a significant risk factor for chronic lung allograft dysfunction.^31^ Finally, chylothorax may occur, notably because many patients with CHD have abnormal lymphatic drainage.^32^

## Technical challenges in CHD repair

Despite technical advances, HLTx remains a very challenging procedure associated with high morbidity and mortality rates compared to isolated lung or heart transplantation, in large part because the main indication is ES.^33^ The main CHDs responsible for ES are ventricular, atrial, and complete atrioventricular septal defect, whose management is not more complex than that of other forms of PH. In contrast, common arterial trunk, patent ductus arteriosus, abnormal inferior vena cava drainage, situs inversus, and aortic coarctation may be more challenging to repair, with graft interposition required in some cases, increasing the risk of bleeding and thromboembolism.^34^ The long required CPB times carry risks of postperfusion syndrome, primary lung-graft dysfunction, and heart-graft dysfunction.^29^ In the ISHLT registry, 1-year mortality was 25%.^2^ However, a marked decrease in mortality has been reported recently by expert centers. Thus, in 1 study, survival in 2001-2022 was significantly higher than in 1981-2000 at 30 days (94% vs 85%; *p* = 0.025) and 1 year (87% vs 74%; *p* = 0.0069). Conditional 10-year survival (in patients alive at 1 year) was similar in the 2 groups, suggesting advances in early postoperative management as the cause of improved survival.^9^ However, graft dysfunction seems more common after HLTx than after lung or heart transplantation. ISHLT data indicate higher rates of early graft failure in HLTx recipients (6.8% vs 2.3%).^2^, ^29^ To avoid graft failure, CHD repair with lung transplantation may deserve consideration, although poorer outcomes compared to HLTx have been reported.^35^ Close collaboration between expert thoracic and CHD cardiac surgeons is crucial, and complex procedures should be reserved for high-volume expert centers. HLTx should always be preferred over DLTx combined with CHD repair.

## Bridging strategy

In end-stage heart and/or lung disease, extracorporeal membrane oxygenation (ECMO) may serve as a bridge to transplantation.^36^ VA-ECMO bridging provides both oxygenation and mechanical support. However, data are limited, and the potential benefits of preoperative ECMO as a bridge to HLTx in adults remain unclear. Prolonged ECMO is associated with coagulopathy that increases the bleeding risk and induces complications associated with limited ambulation.^37^ However, UNOS data on HLTx recipients in 1995-2011 show poorer outcomes in patients who required pretransplantation iMV or ECMO than in controls (1-month survival: 20% vs 83.5%; and 5-year survival: 20% and 45.4%, respectively).^38^ These data have led some institutions to contraindicate HLTx in patients on VA-ECMO. We discourage ECMO bridging to HLTx in patients with ES.

## Perioperative management

Prevention of intraoperative bleeding must start at the time of organ retrieval. Back bleeding from the graft must be avoided and meticulous hemostasis of the donor posterior mediastinum must be performed during heart-lung-block procurement. The mediastinal tissue is carefully ligated on the graft side. The ligatures are placed in contact with the esophagus and the descending aorta to optimally preserve the bronchial blood supply.^26^ For the recipient, a clamshell incision provides access to the posterior mediastinal tissue and heart-lung block. Dissection should be minimal. In many cases, excision of the residual pulmonary artery, left atrium, and pulmonary veins exposes more collaterals and is therefore counterproductive.^27^ Minimizing excision of the residual pulmonary artery also prevents injury to the left recurrent laryngeal nerve. To avoid coagulopathy and minimize the anticoagulant therapy required by CPB, most of the dissection should be performed before CPB initiation.^26^ New hemostatic energy-based devices can help perform bloodless dissection. Ultrasonic scalpels designed to coagulate vessels up to 7 mm in diameter provide hemostasis and shorten the operative time.^39^ Argon beam coagulators can help control bleeding, even when massive and in anticoagulated patients.^40^ After ultrasonic scalpel dissection, chest-cavity hemostasis can be completed using an argon beam coagulator. Mechanical stapling provides complete hemostasis for extrapericardial pneumonectomies and creation of a passage for the transplanted right lung.^27^ In the most severe cases, perioperative systemic artery embolization can be performed. In ES with arterial collateral development, bronchial-artery embolization has been used successfully to stop hemoptysis.^25^, ^41^ Intercostal- and chest-artery embolization effectively stops bleeding in several indications and can be used before transplantation to decrease the risk of perioperative bleeding.^42^ A modified HLTx technique designed to avoid nerve and lymphatic injuries involves extrapericardial pneumonectomies without removal of the trans-pericardial portion of the pulmonary artery and veins, to protect the phrenic and vagus nerves.^26^ The limited dissection around the left pulmonary artery also helps prevent injury to the left recurrent laryngeal nerve. Moreover, the phrenic nerves are not mobilized on pedicles, and fenestrations are created posterior to the phrenic nerves. The heart-lung block is placed posterior to the phrenic nerves, allowing easier rotation of the graft to address any posterior bleeding even after the anastomoses have been completed. However, phrenic nerve dysfunction and posterior mediastinal bleeding still constitute challenges.

In conclusion, perioperative bleeding is a major risk in HLTx for ES, due to vascular collaterals and previous surgery. All available methods for minimizing perioperative bleeding should be used, including perioperative embolization, mechanical stapling, and the use of an ultrasonic energy-based device and argon beam coagulator. Chest dissection should be minimal to avoid phrenic nerve injury.

## Postoperative management and complications

Postoperative care is similar after HLTx as after DLTx or single-lung transplantation (SLTx), as the lungs, not the heart, are the main site of complications, including infections and acute and chronic rejection.^29^ The abundant blood supply to the tracheal anastomosis via the donor coronary arteries prevents the anastomosis complications that can occur after DLTx. The therapeutic immunosuppression after HLTx is comparable to that after DLTx or SLTx, that is, usually more profound than after heart transplantation. Acute cellular rejection of either the heart or the lungs is less common after HLTx than is rejection of isolated heart or lung grafts.^43^ Of note, acute cellular rejection involves the lungs more often than the heart. Infections also usually affect the lungs and are comparable in frequency to those recorded after DLTx or SLTx. In HLTx recipients, the curative and prophylactic treatment of infectious complications is the same as after lung transplantation. HLTx may be followed by chronic rejection of the heart, the lungs, or both. Coronary artery vasculopathy (CAV) is less common than bronchiolitis obliterans syndrome (BOS) after HLTx.^43^ Thus, in 1 study, rates of CAV 1, 3, 5, and 10 years after HLTx were 3%, 7%, 9%, and 27%, respectively, compared to 8%, 27%, 42%, and 62% for BOS.^44^

## Survival and prognostic factors in heart-lung transplantation

Overall mortality after HLTx has decreased, probably due to a combination of factors such as progress in surgical techniques, improved organ-preservation solutions, the use of tacrolimus for immunosuppression, and long-term follow-up strategies.^2^, ^9^, ^29^ Median survival in the ISHLT registry was 6.5 years in recent years.^2^ However, many deaths occur early after transplantation, and median survival in patients alive at 1 year was 12.8 years.^2^ In a high-volume US cardiopulmonary transplant center, median survival improved to 10.24 years in the 2010s, whereas the corresponding value for isolated lung transplantation was 6 years.^9^ Median survival after isolated heart transplantation during the same period was 12.1 years in the ISHLT registry.^45^ Of note, children, particularly those with CHD, have achieved better survival than adults, possibly reflecting differences in comorbidities and earlier referral. Factors that significantly influence post-transplantation outcomes include donor age, underlying diagnosis, use of pretransplant iMV, cytomegalovirus mismatch, and institutional experience.^2^, ^9^

## The protective role of heart-lung transplantation on chronic dysfunctions

HLTx may protect against the development of BOS and CAV compared to isolated organ transplantation.^43^, ^46^ BOS, characterized by chronic inflammation and fibrosis leading to progressive airway obstruction, affects up to 50% of lung transplant recipients within 5 years, compared to less than one-third of HLTx recipients.^2^ CAV, a major limitation to long-term survival after heart transplantation, is an accelerated form of coronary artery disease characterized by concentric fibrous intimal hyperplasia along the length of coronary vessels.^47^ Multiorgan heart transplantation is associated with a lower incidence of CAV.^48^ Specifically, the 5-year incidence of CAV was 33% in isolated heart-transplant recipients compared to 27% in multiorgan-transplant recipients, with differences observed as early as the first year post-transplantation. Multiorgan heart transplantation was associated with lower likelihoods of CAV (odds ratio, 0.19; 95% confidence interval, 0.07-0.51; *p* = 0.0009) and of acute cardiac rejection (odds ratio, 0.18; 95% confidence interval, 0.06-0.55; *p* = 0.003).^48^

The mechanisms underlying these protective effects of multiorgan transplantation are unclear. Conceivably, transplanting both a heart and lungs may induce a more tolerogenic immune environment, thereby reducing the incidence of both BOS and CAV. Further research into these protective mechanisms may suggest novel preventive and therapeutic strategies to minimize the incidence and severity of BOS and CAV in all transplant recipients.

## Author contributions

J.L.P., O.M., L.S., E.F.: Conception and design of the study. J.I., J.L.P., G.D., S.H., E.V., O.M., J.N.A., S.D., T.G., L.S., E.F.: Acquisition of data. J.I., J.L.P., S.H., E.V., T.G., L.S., E.F.: Analysis and interpretation of data.

## Disclosure statement

The authors declare that they have no conflict of interest.

Funding: None.
